# Insect Antimicrobial Peptides: Advancements, Enhancements and New Challenges

**DOI:** 10.3390/antibiotics12060952

**Published:** 2023-05-24

**Authors:** Matteo Dho, Valentina Candian, Rosemarie Tedeschi

**Affiliations:** Dipartimento di Scienze Agrarie, Forestali e Alimentari (DISAFA), University of Torino, Largo P. Braccini 2, 10095 Grugliasco, Italy; matteo.dho@unito.it (M.D.); valentina.candian@unito.it (V.C.)

**Keywords:** immune responses, diet, AMPs, antibiotic resistance, mechanism of action, insect rearing

## Abstract

Several insects are known as vectors of a wide range of animal and human pathogens causing various diseases. However, they are also a source of different substances, such as the Antimicrobial Peptides (AMPs), which can be employed in the development of natural bioactive compounds for medical, veterinary and agricultural applications. It is well known that AMP activity, in contrast to most classical antibiotics, does not lead to the development of natural bacterial resistance, or at least the frequency of resistance is considered to be low. Therefore, there is a strong interest in assessing the efficacy of the various peptides known to date, identifying new compounds and evaluating possible solutions in order to increase their production. Moreover, implementing AMP modulation in insect rearing could preserve insect health in large-scale production. This review describes the current knowledge on insect AMPs, presenting the validated ones for the different insect orders. A brief description of their mechanism of action is reported with focus on proposed applications. The possible effects of insect diet on AMP translation and synthesis have been discussed.

## 1. Introduction

**Figure 1:**
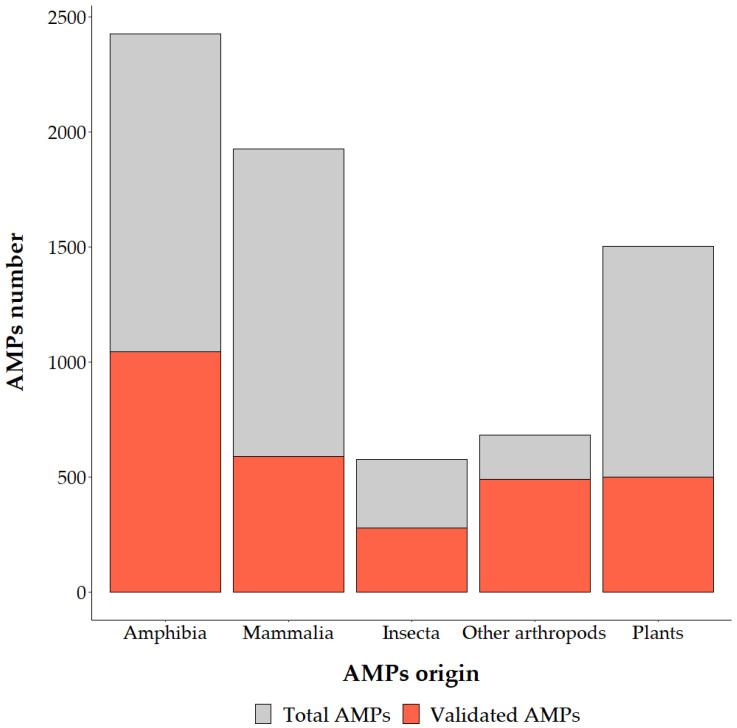
Sources of AMPs based on the data available in dbAMP.

Antimicrobial Peptides (AMPs) are a class of small peptides, usually less than 10 kDa, which play a key role in the innate immune response. AMPs are found in phylogenetically distant organisms, varying from eukaryotes to prokaryotes [1,2]. Even if these molecules are so widespread in nature, animals represent the main source of AMPs studied so far [3,4], and among them, insects are one of the richest reservoirs [5]. To date, the dbAMP database [https://awi.cuhk.edu.cn/dbAMP/, accessed on 17 March 2023] reported 2427 AMPs which have been isolated from amphibians, 1925 from mammals, 1503 from plants, 578 from insects and 682 from other arthropods (Euchelicerata and Malacostraca) (Figure 1) [4]. 

With nearly one million species described, insects are an important source of bioactive compounds [3,6]. Despite the lack of an adaptive immune system, several species can live in highly contaminated substrates and effectively face immune threats. This is possible thanks to the highly effective innate immune response, and AMPs are a crucial part of it [7,8,9,10]. As reported by the Collection of Antimicrobial-Peptides R3 database (CAMP R3), among arthropods, the largest number of AMP families was found in insects [11]. Of the 14 AMP families identified, Abaecins, Cecropins, Coleoptericins, Formaecins, Mastoparans, Metchnikowins and Termicins are exclusive to insects [11] (Table S2). Some examples of high quantity and variability of expressed AMPs are the ladybug *Harmonia axyridis* (Pallas) (Coleoptera: Coccinellidae) with more than 50 putative AMPs [12], the ant *Neoponera goeldii* (Forel) (Hymenoptera: Formicidae) capable of producing more than 15 different Ponericins [13] or *Hermetia illucens* (L.) (Diptera: Stratiomyidae) with 57 predicted genes [9].

**Figure 2:**
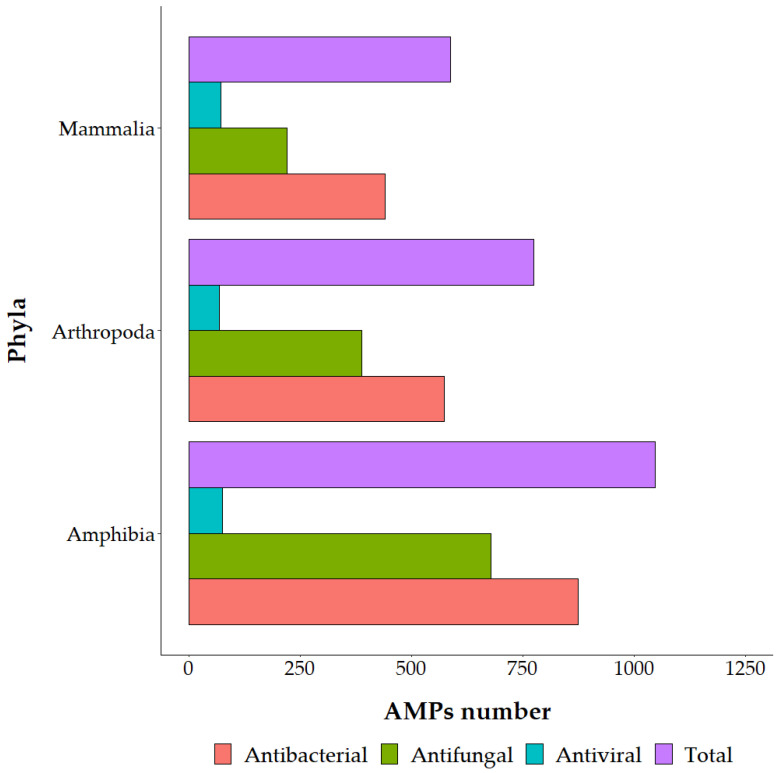
Antibacterial, antifungal and antiviral activities of validated AMPs in different Phyla of the Animalia Kingdom based on the data available in the dbAMP dataset [4].

**Figure 3:**
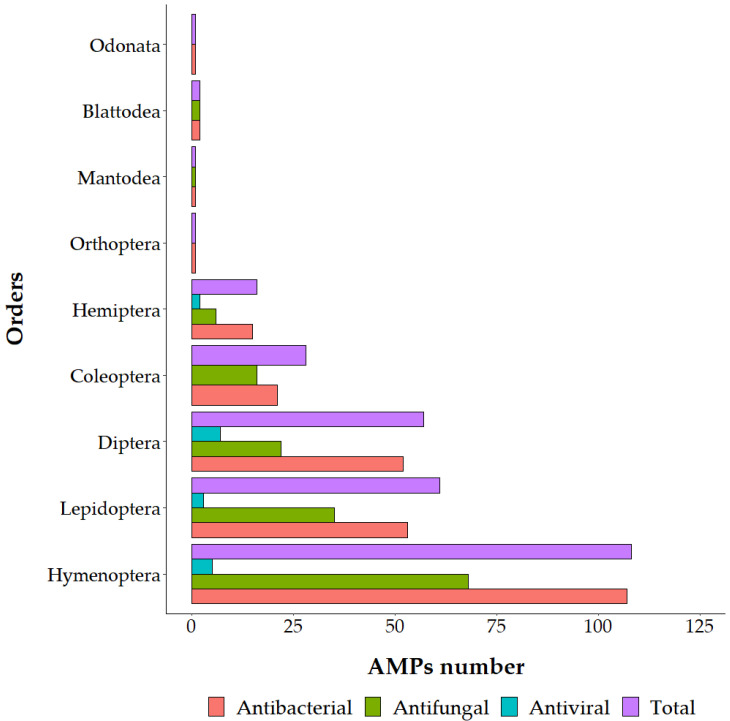
Antibacterial, antifungal and antiviral activities of validated insect AMPs in different insect orders based on the data available in the dbAMP dataset [4].

Since NGS and third generation sequencing are spreading, bioinformatic approaches to identify putative AMPs have been proposed in several papers. From the transcriptome of the grasshopper *Oxya chinensis* subsp. *sinuosa* Mistshenko (Orthoptera: Acrididae), 26 novel AMP sequences were identified [14]. Comparison between AMP expression levels of immune challenged and control insects showed differences in most of the genes [14], helping to identify suitable peptides against a specific pathogen. Inferring putative AMPs alone does not give functional information; antimicrobial activity must be tested. To date, the antimicrobial activity has been validated for about 48% of the AMPs identified in insects and the 72% of AMPs identified in other arthropods (Euchelicerata and Malacostraca) [4] (Figure 1 and Table S1). For example, the transcriptome analysis of American cockroach *Periplaneta americana* L. (Blattodea: Blattidae) allowed the identification of 86 putative peptides [10]. The antimicrobial activity of 25 out of them was tested, and 11 displayed interesting results [14]. Even though some AMPs can display low antimicrobial activity, it is noteworthy that, in nature, they act synergistically, and mixtures of molecules should also be studied to limit the risk of resistance development [15]. Compared to mammals or amphibians, insect AMPs are less studied. Moreover, the current knowledge of insect AMPs is mainly narrowed to model insects with a completely sequenced genome [16]. This suggests there is significant undiscovered AMP diversity hidden among the less well-characterized insect species. Most insect AMPs have been identified in five insect orders (Hemiptera, Coleoptera, Diptera, Lepidoptera and Hymenoptera), which include more than 90% of known insect species (Figure 2) [17]. The majority of insect AMPs, such as insect Defensins, Cecropins, proline-rich peptides and Attacins, have been found in more than two insect orders, but Moricins and Gloverins have been identified only in Lepidoptera [5]. AMPs belonging to the same family or even subfamily of compounds but produced by different insects exhibit different activities [11]. This is because AMPs can be classified according to different factors (activity, shape, size and physicochemical properties). The family division, therefore, does not reflect different activities [18]. Figure 2 reports an overview of the antibacterial, antifungal and antiviral activities of validated AMPs in mainly studied Phyla (Mammalia, Arthropoda and Amphibia) belonging to Animalia Kingdom. An analogous overview detailed for insect orders is reported in Figure 3 (Table S1).

Unlike bacteriocins that are ribosomally synthesisezed and undergo post-translational modifications [19], insect AMPs result from the cleavage of propeptides by proteases [16]. Checking for cleavage sites helps to identify AMP coding genes from genome sequences, apart from sequence homology with already studied peptides [16]. A limitation of bioinformatic methods is the difficulty in predicting post-translational modifications possibly present in final active peptides, such as glycosylation [20,21]. Moreover, the analysis of protein domains with structures similar to AMPs can be a way to develop novel antimicrobial agents. The C-terminus of the human platelet factor IV shows an α-helix motif that has been used to develop the artificial AMP C18G [22]. To date, research works have been mainly focused on the identification, mode of action and biomedical and agricultural applications of these molecules [13,23,24,25,26,27,28,29]. In recent years, the research on insect AMPs is moving towards new perspectives. With the increasing interest for alternative protein sources, in addition to the possibility of exploiting the antimicrobial activity of insects as food or feed, there would be the possibility of modulating the production of AMPs through diet for the purpose of safeguarding the health of insects in mass rearing [30,31,32,33]. Furthermore, applications to protect post-harvested fruits and foodstuffs have been proposed [27,34,35]. This review presents the mode of action and some possible applications of insect AMPs, especially in the agricultural field, but also underlines the importance of the diet in preserving and modulating insect immune responses, a topical issue in insect mass rearing.

## 2. Mechanism of Action of AMPs 

The exact mechanism by which AMPs exert their activities is not completely understood. The majority interact with the phospholipid component of the cytoplasmic membrane of the target microbe, leading to membrane permeabilization, cell lysis and death. Others can act without affecting membrane stability, like impairing nucleic acid biosynthesis, translation, cell division and cell wall biosynthesis or inducing apoptosis [36]. 

### 2.1. Pore Formation

Most of the AMPs are amphipathic molecules, positively charged in the hydrophilic portion. This is necessary for the recognition of the primary target, the microorganism’s membrane [1,37]. By treating Defensin 1 of *Tribolium castaneum* (Herbst) (Coleoptera: Tenebrionidae) with the reducing agent dithiothreitol, the peptide loses its activity against multidrug resistant *Staphylococcus aureus* [38]. Moreover, slightly acidic pH increases protonation of positively charged amino acids, thus increasing antimicrobial activity [39]. Another key aspect for AMP activity is the concentration. Before reaching a threshold level, AMPs tend to bind membranes parallelly without killing the microorganism [40]. AMPs interact with microorganism cell membrane through electrostatic interactions [41], thus making it difficult or less frequent for bacteria to develop resistance, unlike conventional antibiotics [42]. 

**Figure 4:**
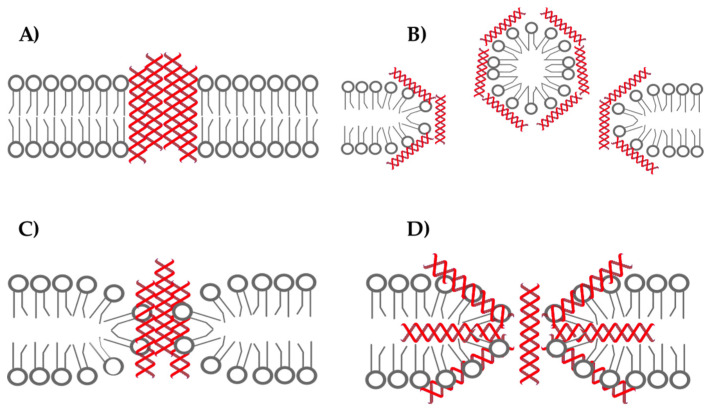
Different models to describe AMP pore-forming activity: (**A**) barrel-stave model; (**B**) carpet model; (**C**) toroidal pore model; (**D**) disordered toroidal pore model.

To describe the activity against membranes, four different models have been proposed so far. Barrel-stave model: after reaching the threshold concentration, AMPs rotate and insert into the membrane bilayer, forming pores. The pore surface is made by the amount of α-helical peptides [43] (Figure 4A). An example of barrel-stave mechanism of action is given by Alamethicin [25]. Carpet model: AMPs with this mechanism tend to accumulate on the membrane surface, with the hydrophilic side associated to the polar head groups. After reaching a proper concentration, the hydrophobic portion rotates towards the hydrophobic chain of the lipid bilayer, creating discontinuity of the membrane in a detergent-like manner [44] (Figure 4B). Dermaseptin S4 follows this mechanism [45]. Toroidal pore model: according to this model, the insertion of multiple molecules leads to a local folding of the membrane, such as the pores are formed by both the AMPs and the folded membrane [40] (Figure 4C). An example is given by Magainin 2 [46]. Disordered toroidal pore model: firstly proposed for Magainin H2 [47]. After reaching a threshold concentration, one peptide rotates and insert into the membrane bilayer, inducing a fast variation in membrane conformation, forming a toroidal shaped pore. The pore is stabilized by the other peptides bound to its edges [47] (Figure 4D).

### 2.2. Other Mechanisms of Action

The pore-forming effect is not the only known mechanism of action. Some AMPs can impede the uptake of thymidine, uridine and histidine in *Escherichia coli*, inhibiting DNA, RNA and protein synthesis [48]. Moreover, Drosocin, Apidaecin and Pyrrhocoricin bind specifically to the chaperons DnaK of *E. coli* but not the human homologue Hsp70, impeding the correct protein folding [49]. Apidaecin binds to the outer membrane of the pathogen before entering the bacterial cell [50]. Temporin L binds to *E. coli* FtsZ, a bacterial tubulin homolog part of the divisome complex, impeding the correct cell division and leading to cell death [51]. C18G inhibits bacterial cell division by activating the PhoQ/PhoP signaling system, which leads to high production of QueE, a protein that associates with the divisome impeding cell division [52].

Furthermore, some AMPs can impede translation. Api137, a derivative of Apidaecin with improved serum stability, binds to the release factors RF1 and RF2 of *E. coli*, preventing ribosome release once reaching the stop codon, causing a general shutdown of the translation process [53]. Other proline-rich insect AMP derivatives, like Oncocin, Metalnikowin I and Pyrrhocoricin, impair translation by binding to the ribosomal A-site, impeding the elongation process [54,55,56].

AMPs can also impair cell wall synthesis. The defensins Lucifensin, Gallicin and Plectasin, the latter of fungal origin, directly binds to the precursor Lipid II of Gram-positive bacteria [57]. Plectasin’s mode of action has been thoroughly studied. It directly binds with the hydrophobic side of the cell membrane, whilst the hydrophilic side binds to Lipid II, impeding its incorporation into the cell wall. Even though there is no pore-forming activity, also in this case, AMP concentration is crucial since the Plectasin–Lipid II binding follows a 1:1 ratio [57]. Several other AMPs from eukaryotic organisms have the same target, such as three oyster Defensins or Copsin from the fungus *Coprinopsis cinerea* [58,59]. Antimicrobial activity of several bacteriocins involves binding cell wall precursors, such as the lantibiotics Nisin, Gallidermin, Mersacidin and Actagardine [60].

Some insect AMPs proved to induce cell apoptosis besides having a pore-forming activity against fungal pathogens. Cecropin A leads to *C. albicans* death by impairing cell ion balance; Ca^2+^ enters the cytosol while K^+^ exits. This ion homeostasis imbalance induces apoptosis and, subsequentially, cell death [61]. Similarly, melittin can induce *C. albicans* death by pore formation and by activating the apoptotic process via metacaspase activation and ROS generation [62]. Metchnikowin toxicity against *Fusarium graminearum* depends on two mechanisms. Metchnikowin impairs cell wall synthesis by targeting the enzyme β(1,3)glucanosyltransferase Gel1, as well as inhibiting succinate dehydrogenase, limiting mitochondrial energetic production [63]. Periplanetasin 4 acts against several yeasts by impairing both vacuolar and mitochondrial homeostasis and communication, increasing superoxide formation and leading to cell death without causing DNA damage [64]. Buda De Cesare et al. [65] reported a thorough overview of antifungal activity.

There are also AMPs with antiviral activity. The human cathelicidin-derived GF-17 can elicit type-I interferon signaling and directly inactivate Zika virus [66]. Melittin and cecropin A can inhibit HIV replication in infected cells by preventing viral gene expression but not host gene expression, without any pore-forming activity [67]. Cecropin D and Cecropin P1 prevent porcine reproductive and respiratory syndrome virus (PRRSV) attachment to the host cell and inhibit host cell apoptosis in late infections in vitro [68,69]. 

## 3. Interactions between AMPs and Cell Wall

**Figure 5:**
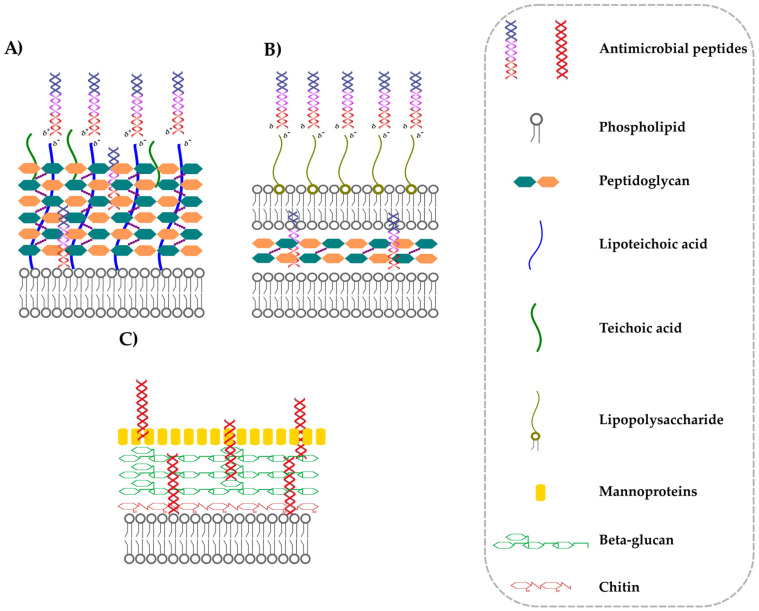
AMP interactions with microorganismal cell wall. (**A**) interaction with Gram-positive bacteria cell wall; (**B**) interaction with Gram-negative cell wall; (**C**) interaction with fungal cell wall.

To express antimicrobial activity, AMPs must also overcome the microorganismal cell wall. Peptidoglycan is not dense enough to impede AMPs from reaching the membrane [70]. Moreover, cell wall precursors can be AMP targets [57,58,59,60,70]. The external side of bacterial cell wall tends to be negatively charged. This net charge can affect AMP activity. Gram-positive bacteria have phosphate groups on the lipoteichoic-acids (LTA) that possibly attract positively charged AMPs, promoting the approach and contact with the cell membrane [71] (Figure 5A). The reduction of negative charge of LTA by D-alanylation or by using NaCl solution increases the resistance of *Staphylococcus* strains against some AMPs, such as Magainin 2 and Colistin [72]. This increased resistance does not depend on the reduced electric interaction but on a different cell wall conformation, resulting in it being denser than the wild type [72]. Similarly, Gram-negative bacteria present a negative net charge on the outer membrane due to lipopolysaccharides (LPS) (Figure 5B). Both the inner- and the outer-core components of LPS deeply affect *E. coli* susceptibility to different AMPs [73]. Loss-of-function mutations of genes responsible for inner-core LPS formation generally increase AMP activity against *E. coli* [73]. The deletion of the outer-core LPS generally favours AMP access to the cell membrane, while its composition can influence the activity of some molecules, such as Melittin, but not Cecropin B and Cecropin P1 [73].

The fungal cell wall harbors receptors essential for the activity of some AMPs (Figure 5C). Plant Defensin NaD1 needs the protein layer of the cell wall to kill *Fusarium oxysporum* f. sp. *vasinfectum* hyphae [74]. The toxic activity of Osmotin, a Thaumatin-like AMP, is enhanced by cell wall components bringing carbohydrates, such as phosphomannoproteins [75]. The Defensin RsAFP2 antifungal activity against *Candida albicans* needs an intact cell wall. RsAFP2 interacts with cell wall glucosylceramides, and loss-of-function mutation of genes involved in cell wall synthesis increases *C. albicans* tolerance to this AMP [76]. Moreover, Heliomicin binds fungal glucosylceramides but not human ones [77]. 

## 4. Proposed Applications of Insect AMPs

Several applications have been proposed for insect AMPs so far, either in the healthcare system, agriculture or food production chain. 

### 4.1. Proposed Applications of AMPs in the Medical Field

Many insect AMPs, such as some Defensins, Thanatins and Coleoptericins, present low to no lithic activity against human erythrocytes [78,79,80]. Multidrug-resistant bacteria (MDR) have become a major healthcare problem, causing more than 35,000 deaths in the USA and approximately 33,000 deaths in Europe per year [81]. For these reasons, insect AMPs and artificial derivatives have been proposed as alternatives or adjuvants to classical antibiotics [82,83,84]. Defensin 1 of *T. castaneum* can inhibit MDR *Streptococcus pneumoniae* growth in vitro, decreasing cytokines production in infected macrophages [85]. The same Defensin 1 is active against methicillin-resistant *S. aureus* strains in vitro, increases infected *Caenorabditis elegans* adult survivors, and shows no cytotoxicity against human erythrocytes [38]. The Defensin TcPaSK, artificial derivative of *T. castaneum* Defensin 3 with higher cationicity and decreased hydrophobicity, can counteract *S. aureus* growth through membrane permeation, but there is evidence of a possible block of cell division [86]. Oxysterlin 1, 2 and 3 from *Oxysternon conspicillatum* are active against MDR bacteria, such as *Klebsiella pneumoniae* and *Pseudomonas aeruginosa*, but generally not against Gram-positive bacteria with limited hemolytic activity [87]. Furthermore, an isoform of Cecropin B called CecB Q53 showed promising ability to contain *P. aeruginosa*, *E. coli* and *Staphylococcus*, with high stability to pH, salinity and temperature variations, showing no significant cytotoxic or hemolytic activity [88].

In addition to exerting a defensive function in immune challenges, an antitumoral activity of insect AMP was assessed. In *Drosophila melanogaster* (Meigen) (Diptera: Drosophilidae), larvae affected by hyperplasia of lymph gland have a higher expression of AMP coding genes [89]. An ectopic expression of defensin, drosomycin and diptericin in the fat body significantly suppressed the LG hyperplasia phenotype in mutants exhibiting the malignan hyperplasia [89]. Focusing on healthcare applications, Cecropin XJ isolated from *Bombyx mori* L. (Lepidoptera: Bombycidae) selectively inhibits gastric cancer cell growth in vitro without harming healthy cell development and reduces cancer growth in mice in vivo [90]. The same AMP also impairs the normal cytoskeletal organization of esophageal carcinoma cells Eca109 but not in healthy cells [91]. Cecropin A and B induce bladder tumor cell lysis in a concentration three times lower than that required to harm healthy cells [92]. Furthermore, synthetic AMPs have been studied for possible antitumoral applications. CA-M peptide is the fusion of the N-terminal regions of Cecropin A and Melittin, known for its antitumoral activity but with high cytotoxicity [93,94]. CA-MA peptide is the fusion of N-terminal regions of Cecropin A and Magainin. Both these peptides inhibit small cell lung cancer cell growth in vitro, with CA-MA performing better in terms of antitumoral and low hemolytic activity [94].

Another property of insect AMPs is the ability to contrast tripanosomatids. The recombinant insect Defensin rDef1.3, derived from a Defensin of *Triatoma pallidipennis* (Stål) (Hemiptera: Reduviidae), actively increases *Trypanosoma cruzi*, *Trypanosoma rangeli* and *Leishmania major* death and altered morphology [95]. Cecropin A is active against the intracellular form of *Leishmania panamensis* [96], while Attacin of *Glossina morsitans morsitans* Westwood (Diptera: Glossinidae) inhibits *Trypanosoma brucei* growth in vitro [97]. Moreover, some AMPs are proven to be spermicidal agents with antimicrobial activity that could possibly prevent sexually transmissible diseases [98].

### 4.2. Proposed Applications of Insect AMPs in Agriculture 

The world’s population is continuously growing and, therefore, the food and feed demand. To ensure adequate production, it is mandatory to protect plants from pathogens [99]. A recent study estimates two million tonnes as the amount of pesticides used worldwide [100]. However, the emergence of resistant pathogens and the need to increase yields leads to requirements of developing environmentally safe alternatives to protect plants [100,101]. Insect AMPs have also been studied for plant protection. For example, transgenic tobacco plants expressing heliomicin or drosomycin in the apoplast showed moderate resistance against *Cercospora nicotianae* [102]. Transgenic barley with apoplastic expression of metchnikowin impedes functional haustorium formation of the ascomycete *Fusarium graminearum* but has a low effect against the basidiomycetes *Piriformospora indica* and *Rhizoctonia solani*, a mycorrhizal and a pathogenic fungus, respectively [103]. Not only herbaceous plants but also *Citrus sinensis* have been transformed with insect AMP coding construct. The expression of attacin A in the apoplast results in significantly lower susceptibility to *Xanthomonas axonopodis* pv. *citri* [104]. The transformation of *Brassica napus* for the expression of cecropin P1 confers resistance to the bacterial pathogen *Erwinia carotovora* and the fungus *Fusarium sporotrichioides* [105]. Interestingly, these transgenic plants also have enhanced resistance to the herbicide Paraquat [105]. 

Few studies have been conducted on applications different from genetic engineering. One example is given by Melittin injected in and sprayed on rice plants. It effectively protects plants from *Xanthomonas oryzae* pv. *oryzae*, mainly by forming pores in the pathogen membrane but also by binding to DNA and RNA intracellularly [106]. Different from the direct application on human tissues, Melittin does not harm rice cells [106]. Treatment with 1 μg and 4 μg of CA-M peptide on potato tuber slices inoculated, respectively, with *Erwinia carotovora* subsp. *atrosepica* and *Erwinia chrysanthemi*, prevent damage formation [107].

Insect AMPs also showed positive health effects in animal farming, resulting in a possible alternative to the wide antibiotics usage [108]. A Cecropin A–Cecropin D hybrid AMP (CAD) tested in broiler rearing managed to improve feed:gain ratio and decrease jejunum villi inflammation [109]. The same peptide was tested in fish rearing. A moderate supplementation of CAD to a standard diet increases *Scophthalmus maximus* survival to inoculation with *Edwardsiella tarda*, whilst a high supplementation does not change fish survival and deeply affects turbot gut microbiota composition [110]. The moderate usage of Cecropin in tilapia diet has a positive effect on fish feed:gain ratio, enhancing innate immune system activation and decreasing mortality after injection with *Aeromonas veronii* [111]. Moreover, in pig breeding, Cecropin AD showed to be a promising alternative to traditional antibiotics in helping weaning piglets face immune challenges and increasing growth performances [112]. The supplementation with Cecropin A in the late laying period of hens significantly increases the number of laid eggs without affecting product quality [113]. Whilst there is a variation in caecal microbiota composition, it only affects less abundant microorganisms, such as Verrucomicrobia and Cyanobacteria, without any clear effect on hens’ health [113].

### 4.3. Proposed Applications of Insect AMPs in the Food Chain

With a constantly growing world population and the increase of pathogen strains resistant to commonly used food preservatives, there is a need for identifying novel food preservatives and developing active packages to increase the shelf life of products. For this purpose, AMPs have been widely studied [35,114,115]. Up to now, the bacteriocin Nisin is the only used and approved AMP in the food industry [116], but research on insect-derived AMPs is getting more interest [34,35,117]. 

Hf-1, a peptide purified from *Musca domestica* L. (Diptera: Muscidae) larvae, showed bactericidal activity against *E. coli*, *P. aeruginosa*, *Salmonella typhimurium*, *Shigella dysenteriae*, *S. aureus* and *Bacillus subtilis*. The antibacterial activity was almost equivalent to sodium benzoate in orange juice [118]. Jelleine-1, normally present in royal jelly, showed strong antimicrobial activity against food-borne *Listeria monocytogenes*, with both a pore-forming activity and deleterious interaction with pathogen DNA. Furthermore, biofilm formation is strongly impaired by Jelleine-1 [119]. 

Since AMPs act synergistically in natural conditions, the use of insect extracts containing AMPs has also been proposed. A mix of nineteen AMPs belonging to seven different peptide families extracted from *M. domestica* pupae showed promising preservation on chilled pork meat [34]. An extract from *T. molitor* larvae challenged with edible *Lactiplantibacillus plantarum* rich in AMPs displayed high antimicrobial activity against food-borne bacteria and aflatoxin-producing fungal strains. This extract shows high stability in a wide range of temperature, salinity and pH, resulting as an optimal soy sauce preservative [117]. Apart from food preservation, AMPs have also been proposed for active packaging production [35,115].

## 5. Effect of Insect Diet on AMP Translation and Synthesis

In mass rearing of insects intended for AMP extraction or to produce peptide-rich insect products, it is necessary to identify strategies to increase their content. To date, this is achieved by infecting insects with microorganisms. Immune challenge to increase AMP production presents important drawbacks. It has been shown that, in immune challenged *D. melanogaster*, the fat body metabolism switches from an anabolic to a catabolic response, with energy redirected to immune response [120]. Furthermore, the normally phosphorylated transcription factor MEF2 activates anabolism-related genes in fat bodies. During an immune response, MEF2 dephosphorylation results in the activation of AMP coding genes [121]. The energy reallocation from anabolism to insect immune responses leads to a lower insect growth that may be deleterious in some insect productions (e.g., when the AMP extraction is not the only production of interest). Recent studies report changes in the expression of AMP encoding genes after rearing diet modulation alone and not necessarily following an immune challenge [30,31,32,33]. Interestingly, these variations showed to be more beneficial than the addition of bacteria to standard diet [30].

Since diet modulation is leading to promising positive results for both immune response regulation and insect growth, diet optimization is also becoming a topical issue to counteract disease outbreaks in large-scale production [122]. The diet-mediated effect on immunity is still poorly investigated both in useful and pest insects. Diet composition affects insect physiology and nutritional composition [123,124,125]. The diet protein content directly influences the production of amino acids that can be used to synthetize substrates and enzymes involved in defence reactions [126]. Nutrient-poor diets and non-optimal protein/carbohydrate ratios result in a lower production of AMPs [30,127]. *Tenebrio molitor*, *Spodoptera littoralis* (Boisduval) (Lepidoptera: Noctuidae), *Spodoptera exempta* (Walker) (Lepidoptera: Noctuidae), as well as other insects facing an immune threat prefer to ingest high protein feed [128,129,130,131,132,133,134,135]. *Apis mellifera carnica* Pollmann (Hymenoptera: Apidae) fed protein diet enriched with *Sinapis* spp., *Asparagus* spp. and *Castanea* spp. monofloral pollen showed higher apidaecin 1 and abaecin coding gene expression level [136]. Brewers spent grain-added diet leads to increased AMP expression in *H. illucens* and *T. molitor* rearing [30,33].

Different diets showed to deeply affect AMP transcription in *H. illucens* [30,31,32]. Higher AMP transcriptional levels have been observed in insects reared on catering waste [32] due to the great microbial load of the substrates [137,138]. The addition of sunflower oil to a standard diet increased AMP transcription [30,31]. The inhibitory activity of insect extracts against two Gram-positive and two Gram-negative bacteria confirmed molecular data [30]. The stimulation of the immune system may be due to the vegetal oil composition and the presence of phytosterols [30]. It is documented that phytosterol-enriched diets increase AMP transcription of *Helicoverpa zea* (Boddie) (Lepidoptera: Noctuidae) larvae. The uptake and incorporation of phytosterols into insect cell membranes lead to membrane alterations and leaky cells. This, in turn, could lead to a recognition of self-damage or induction of general stress responses resulting in the upregulation of AMP coding genes after feeding [139]. Moreover, the timing of diet administration showed to influence AMP transcriptional levels [31,33] probably due to different metabolic receptivity of the different larva instars. In addition, diet composition also affects gut microbiota, which plays a substantial role in insect immune system regulation [140,141].

Not only composition but also the abundance of feeding substrate is crucial for the survival of immune-challenged insects [142,143]. *Galleria mellonella* L. (Lepidoptera: Pyralidae) larvae deprived of food for seven days demonstrated increased susceptibility to infection by *C. albicans* due to cellular and humoral response reduction. Starving leads to a reduction in the expression of a variety of AMPs and immune proteins. Lipocalin coding gene expression was reduced by approximately 14% in starved larvae [140,141].

A deep correlation between immune and metabolic responses has been shown in *D. melanogaster*. The infection with Gram-positive bacteria activates the Toll-pathway, which enhances AMP transcription but also inhibits insulin receptors [144]. Interestingly, the presence of insulin also modulates AMP transcription. The insulin pathway inactivates the transcription factor Forkhead box O (FOXO). Several AMP coding genes have FOXO binding sites, such as the drosomycin gene [145]. Recently, investigators have identified the role of nutrient-sensing pathways, particularly the insulin signaling (ILS) pathway, in regulating components of the immune response [46,47]. A further link between diet and AMP production was assessed for *step* mutants, an essential component of insulin/insulin-like growth factor signaling (ILS), and *chico* mutants (i.e., the insulin receptor). In both cases, increased production of AMPs was obtained in non-infected insects [145].

Although the rearing diet could positively influence AMP transcription, it does not always correspond to a higher translation. Biological assays or analyses of specific AMPs are needed to confirm it [132]. Indeed, there are numerous steps between gene expression and AMP production, and multiple factors can affect this process. Upregulation of gene expression is expected to change the abundance of proteins concordantly, but there can be a time lag between mRNA levels and protein abundance [146,147]. Some genes are constitutively transcribed, but protein translation only occurs when the correct conditions are met [148]. When amino acids are limiting, gene transcription still occurs, but the gene may not be translated until their availability increases [149].

The possibility to modulate AMP production with the diet opens new perspectives in the industrial rearing of insects as food and feed. On the one hand, it can be an important tool to preserve the health of the insects, avoiding the use of antibiotics and preventing mass death with important economic losses. On the other hand, it launches the use of insects as food or feed not only in terms of nutritional value but also as a novel ingredient that can improve the health of humans and livestock with immunity-enhancing effects. In particular, a diet-dependent expression of AMPs translated into diet-dependent profiles of inhibitory activities against a spectrum of bacteria opens a new and important chance to transfer these AMPs in the final consumers.

## Data Availability

The data presented in this study are available in the Supplementary Material.

## Supplementary Materials

The following supporting information can be downloaded at: https://www.mdpi.com/article/10.3390/antibiotics12060952/s1, Table S1: validated insect AMPs downloaded from dbAMP; Table S2: dataset of AMPs downloaded from CAMP_R3_.
